# Comparison of efficacy of robotic surgery, laparoscopy, and laparotomy in the treatment of ovarian cancer: a meta-analysis

**DOI:** 10.1186/s12957-019-1702-9

**Published:** 2019-09-13

**Authors:** Can Shi, Yingchun Gao, Yijun Yang, Lei Zhang, Juanpeng Yu, Ting Zhang

**Affiliations:** Department of Obstetrics and Gynecology, The Affiliated Huaian No. 1 People’s Hospital of Nanjing Medical University, No. 1 Huanghe West Road, Huaiyin District, Huai’an, 223300 Jiangsu China

**Keywords:** Ovarian cancer, Robotic surgery, Laparotomy, Laparoscopy, Minimally invasive surgery

## Abstract

**Background:**

We intended to compare the clinical effect of robotic surgery with laparoscopy and laparotomy in ovarian cancer treatment.

**Methods:**

The included studies were retrieved from PubMed, Embase, and the Cochrane Library databases. The Methodological Index for Nonrandomized Studies (MINORS) was used to evaluate the study quality. Effect measures were presented with weighted mean difference (WMD)/odds ratio (OR) and 95% confidence interval (CI), and heterogeneity test was assessed using *Q* test and *I*^2^ statistics to determine the use of the random effects model or fixed effects model. Egger’s test was used to assess the publication bias.

**Results:**

A total of eight studies was included in this meta-analysis with a MINORS score of 16–18. In the random effects model, estimated blood loss (EBL) of robotic surgery was significantly less compared with laparotomy (WMD = − 521.7027, 95% CI − 809.7816; − 233.6238). In the fixed effects model, length of hospital stay (LHS) (WMD = − 5.2225, 95% CI − 6.1485; − 4.2965) and postoperative complication (PC) (OR = 0.4710, 95% CI 0.2537; 0.8747) of robotic surgery were significantly less, and overall survival (OS) rate (OR = 6.4355, 95% CI 1.6722; 24.7678, *P* = 0.0070) of robotic surgery was significantly higher compared with laparotomy. There was no difference in the effect size of all variables between robotic surgery and laparoscopy. Meanwhile, a publication bias (*t* = 6.8290, *P* = 0.002405) was only identified for PC in robotic surgery and laparotomy groups; no publication bias was identified for the other variables.

**Conclusions:**

Despite the above results, it failed to show oncological safety and recurrence by pathological stages or histologic types in this meta-analysis, and those confounding factors might affect the clinical outcome. Future meta-analyses with a larger number of eligible randomized controlled trial studies were needed to determine the most suitable treatment method for patients with different stages and types of ovarian cancer.

**Electronic supplementary material:**

The online version of this article (10.1186/s12957-019-1702-9) contains supplementary material, which is available to authorized users.

## Background

Ovarian cancer is one of the most common gynecological carcinomas with high fatality and recurrence rate [1]. The early diagnosis of ovarian cancer is extremely difficult due to locating deep in the pelvic cavity and lacking of typical symptoms, and most patients are preliminarily diagnosed as advanced stage with a bad prognosis and 5-year survival rate [2]. The basic treatment of ovarian cancer is still the traditional radical surgery combined with adjuvant chemotherapy. The surgery is performed to obtain a pathological diagnosis and to resect tumor tissue [3, 4].

With the development of minimally invasive surgery, the reports for laparoscopic surgery in ovarian cancer present an increasing trend. Laparoscopy provides an intuitive examination into the pelvic and abdominal organs, which is as effective as traditional laparotomy for the diagnosis of ovarian cancer with avoiding unnecessary laparotomy [5, 6]. The laparoscopy staging is an exact staging method of ovarian cancer with lesser complications and shorter hospital stay compared with laparotomy [7]. Laparoscopy provides multiple advantages in the treatment of early ovarian cancer, such as minimal abdominal incision and a decrease of recovery time. Meanwhile, there remain some disadvantages for laparoscopy in the treatment of early ovarian cancer, including preventing palpation of lymph nodes and existing a possible risk of trocar site metastasis [8].

Robotic surgery is an emerging technology in minimally invasive surgery, which overcomes some barriers of traditional laparotomy and laparoscopy. Such as, it provides three-dimensional (3-D) visualization instead of two-dimensional (2-D) visualization in laparoscopy; it remains stable and tireless which avoids the doctor’s physiologic tremor and fatigue in laparotomy; in addition, it enhances dexterity, degree of freedom, and instrument motion [9]. Based on the multiple advantages, robotic surgery presents an ever-increasing trend in gynecological oncology. Reportedly, compared with traditional laparotomy and laparoscopy, the robot surgery is viable and secure in cervical cancer therapy with less bleeding and complication as well as shorter recovery time [10]. Robotic system is secure and effective in gynecology and gynecological oncology, and it may be more fit for operating in confined spaces with difficult access, like pelvic cavity [11]. A previous review has reported that robotic surgery is expected to turn into the preferred surgical method in gynecological oncology, but it still needs to be confirmed by the results of comparing with traditional laparotomy and laparoscopy [12]. In the current study, a meta-analysis was performed to compare the effect of robotic surgery, laparoscopy, and laparotomy in the treatment of ovarian cancer, which was expected to confirm the clinical value of robotic surgery.

## Methods

This meta-analysis was carried out in accordance with the Preferred Reporting Items for Systematic Review and Meta-analysis (PRISMA) guidelines [13].

### Search strategy

The relevant clinical studies were retrieved from the PubMed (http://www.ncbi.nlm.nih.gov/pubmed), Embase (http://www.embase.com), and the Cochrane Library (http://www.cochranelibrary.com) databases with search terms as “Robot*” OR “Da Vinci” OR “Leonardo's” AND “ovarian cancer” OR “oophoroma” OR “carcinoma of ovary” OR “ovarian carcinoma” OR “ovary cancer” AND “Laparoscopy” OR “laparoscopic” OR “laparotomy” OR “Open surgery” (up to November 15, 2018). The detailed retrieval strategies from PubMed are shown in Additional file 1: Table S1.

### Inclusion and exclusion criteria

The literature screening was performed by two investigators, and the disagreements were settled by discussing with the third investigator. The literatures were selected with the following criteria: all the patients were women with ovarian cancer; the studies contained the robotic surgery group (patients with robotic surgery treatment) and the control group (patients with laparoscopy or laparotomy treatment), and the outcomes included overall survival (OS) rate, disease-free survival (DFS) rate, estimated blood loss (EBL)/ml, length of hospital stay (LHS)/days, operating time (OT)/min, postoperative complication (PC), pelvic nodes (PN), and postoperative recurrence (PR) or included at least one of them; the patients with or without neoadjuvant chemotherapy were all included; the types and language for the studies had no restriction. Meanwhile, the literatures with the following criteria were excluded: (1) data were incomplete and could not be used for statistical analysis; (2) studies were reviews, letters, and comments, etc.; (3) among repeated studies or multiple studies involving the same data, only the latest or the one with the most detailed information was selected; (4) there were less than five patients in each group.

### Data extraction and quality evaluation

The data extraction and quality evaluation were also carried out by two investigators, and the disagreements were settled by discussing with the third investigator. The extracted information in each study included first author’s name, year of publication, study time, area and type, follow-up time, stage of ovarian cancer, the number of patients, age, body mass index (BMI), and the outcomes (including EBL, LHS, OT, PC, PN, and PR). The Methodological Index for Nonrandomized Studies (MINORS) [14] was used to conduct the quality evaluation for the studies, and the MINORS contained 12 items, such as a clearly stated aim, inclusion of consecutive patients, prospective collection of data, and endpoints appropriate to the aim of the study. Each item was scored from 0 to 2, and the total score was 24. The items with score = 0 represented that the study was not reported, and the items with score = 1 indicated that the study was reported with insufficient information, and the items with score = 2 indicted that the study was reported with sufficient information.

### Statistical analysis

The R 3.12 software (R Foundation for Statistical Computing, Beijing, China, “meta” package) was used to carry out the meta-analysis, and the effect measures were presented with weighted mean difference (WMD)/odds ratio (OR) and 95% confidence interval (CI) [15]. Heterogeneity between studies was assessed using *Q* test [16] and *I*^2^ statistics [17], and *P* < 0.05 or *I*^2^ > 50% were regarded as statistically significant. The random effects model was used to pool the estimates when there was a significant heterogeneity between studies; otherwise, the fixed effects model was selected. The Egger’s test [18] was utilized to evaluate the publication bias. In addition, the sensitivity analysis was performed by comparing the pooled WMD/OR values and their 95% CI in random effects model and the fixed effects model, and the consistent estimates in the two models indicated the stable conclusions.

## Results

### Study retrieval

**Fig. 1:**
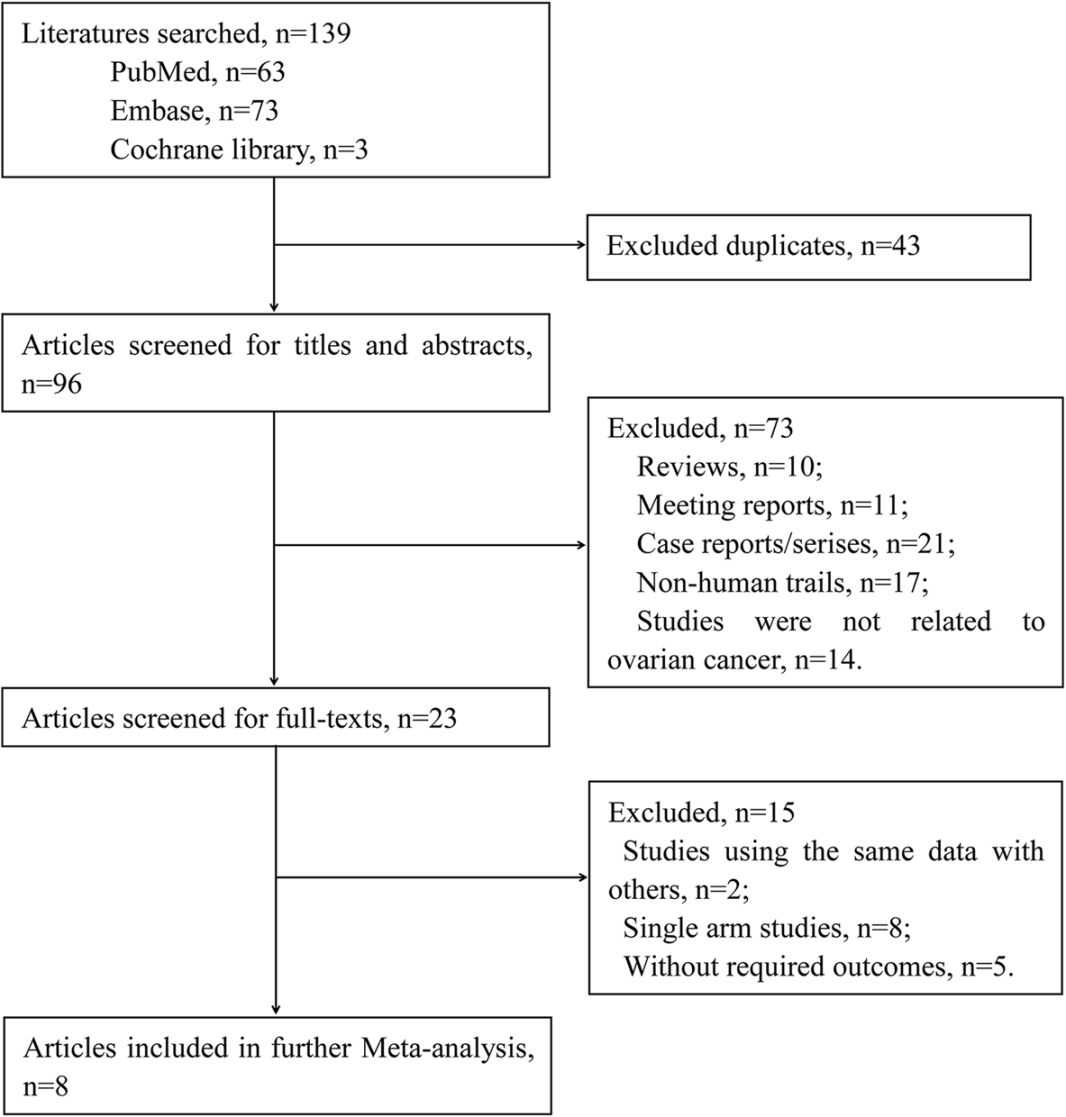
Flow of literature search and study selection

In all, 139 studies were retrieved from the PubMed, Embase, and Cochrane library databases based on the predefined search strategy, and 23 studies were retained after removal of 43 repeated studies, 10 reviews, 11 conference reports, 21 case reports/series, 17 non-human studies, and 14 irrelevant studies. Then, 15 studies were excluded according to full-text reading, including 8 studies about single-arm, 2 studies involving the same data, and 5 studies without available statistical data. Finally, 8 studies [19–26] were selected and performed the following meta-analysis. The detailed filtering process is shown in Fig. 1.

### Characteristics of studies

A total of 647 patients with ovarian cancer were registered in the eight included studies, including 207 patients with robotic surgery treatment and 166 patients and 274 patients with laparoscopy and laparotomy treatment, respectively. The detailed characteristics of the 8 included studies are shown in Table 1. All the studies were carried out in Italy, China, the USA, or Taiwan from 2005 to 2016 and published during a period from 2011 to 2017. The study type was non-randomized clinical study, and the duration of follow-up was 12–54 months. Most patients were middle-aged and elderly with an average age among 43–67 years and suffered an early stage of ovarian cancer. In the study of Feuer et al., there are 52% and 15% patients in the R group and O group accepting neoadjuvant chemotherapy, respectively. In the study of Ye et al., there are 2 patients accepting neoadjuvant chemotherapy, and there is no relevant information in other studies. The average BMI value was between 22.3 and 28.2, and there was no difference between groups in each study. The outcome variables (EBL, LHS, OT, PC, PN, and PR) in the eight studies are shown in Table 2.

**Table 1:** Characteristics of included literature

Author	Public year	Study year	Location	Stage	Type of tumor	Residual disease ^#^	Group	*N*	Age, years*	BMI (kg/m^2^)*	Follow-up, months
Ye et al. [26]	2017	2014.11–2015.11	China	I	NA	NA	R	9	45.7 ± 13.8	24.4 ± 4.4	12–24
							L	10	NA	NA	12–24
							O	8	NA	NA	12–24
Gallotta et al. [23]	2016	2014.10–2016.4	Italy	IA–IIIB	NA	NA	R	32	49 (32–76)	24 (17–54)	Median 38
							L	64	49 (27–73)	24 (19–41)	Median 38
Bellia et al. [19]	2016	2006–2014	France, Italy	IA–IIIC	Epithelial	NA	R	16	47.3 ± 12.3	22.3 ± 2.9	21.2 ± 12.7
							L	23	49.4 ± 15.9	25.8 ± 6.5	18.5 ± 8.6
Chen et al. [21]	2015	2007.9–2015.2	Taiwan	Recurrent	NA	NA	R	8	56.3 ± 12.4	25.9 ± 5.6	NA
							L	12			NA
							O	15			NA
Magrina et al. [24]	2011	2004.3–2008.12	USA	I–IV	Epithelial	21	R	25	65 (23–82)	25.1 (18.7–35.8)	30
						25	L	27	59 (3–85)	23.7 (17.6–37.7)	54
						67	O	119	67 (19–90)	24.9 (16.6–38.5)	42
Feuer et al. [22]	2013	2008–2012	USA	I–IV	Epithelial	46	R	63	59.8 ± 11.8	27.1 ± 7.3	15.5 ± 12.3
						13	O	26	55.7 ± 11.7	28.2 ± 6.1	23.5 ± 14.0
Magrina et al. [25]	2013	2006.1–2010.12	USA	Recurrent	NA	7	R	10	66.0 (45.0–83.0)	22.6 (19.7–25.5)	NA
						8	L	9	57.0 (45.0–74.0)	24.5 (19.9–2.7)	NA
						24	O	33	62.0 (39.0–80.0)	25.9 (18.5–41.2)	NA
Chen et al. [20]	2016	2005–2014.	Taiwan	IA–IIIC	Epithelial	44	R	44	44.3 ± 12.3	22.3 ± 2.7	13.1 ± 5.3
						21	L	21	43.8 ± 10.3	24.1 ± 4.9	29.6 ± 19.0
						72	O	73	49.2 ± 12.8	22.9 ± 4.2	26.7 ± 17.7

*R* robot, *L* laparoscopic, *O* laparotomy, *BMI* body mass index, *NOS* Newcastle-Ottawa Scale, *NA* data are not available

^#^Number of complete debulking

*Mean ± SD/median (range)

**Table 2:** Distribution of extracted data

Author	Year	Group	*N*	EBL, ml*	LHS, days*	OT, min*	PC	PN*	PR	OS rate	DFS rate
Ye et al. [26]	2017	R	9	208.9 ± 202.7	11.1 ± 3.5	251.4 ± 58.7	0	27.8 ± 8.9	NA	NA	NA
		L	10	179.0 ± 234.0	15.8 ± 6.6	233.5 ± 75.9	1	27.3 ± 9.4	NA	NA	NA
		O	8	375.0 ± 353.6	13.1 ± 4.6	226.0 ± 69.8	2	25.6 ± 7.0	NA	NA	NA
Gallotta et al. [23]	2016	R	32	NA	NA	NA	1	NA	NA	NA	NA
		L	64	NA	NA	NA	4	NA	NA	NA	NA
Bellia et al. [19]	2016	R	16	NA	NA	270 ± 72	4	NA	1	16	15
		L	23	NA	NA	288 ± 88	3	NA	2	22	21
Chen et al. [21]	2015	R	8	68.7 ± 53	4.8 ± 2.7	NA	1	NA	NA	NA	NA
		L	12	95.8 ± 45	8.4 ± 8.2	NA	1	NA	NA	NA	NA
		O	15	256.4 ± 258.5	16.2 ± 16.8	NA	6	NA	NA	NA	NA
Magrina et al. [24]	2011	R	25	164 ± 113	4 ± 3	315 ± 102	6	11.7 ± 6.9	NA	NA	NA
		L	27	267 ± 300	3 ± 2	254 ± 83	1	13.9 ± 4.9	NA	NA	NA
		O	119	1307 ± 1060	9 ± 7	261 ± 77	40	13.6 ± 7.1	NA	NA	NA
Feuer et al. [22]	2013	R	63	94.9 ± 72.9	2.3 ± 3.0	138.6 ± 38.7	10	13.3 ± 7.9	15	60	NA
		O	26	385.4 ± 219.4	6.2 ± 4.9	95.2 ± 31.3	6	10.7 ± 6.8	5	19	NA
Magrina et al. [25]	2013	R	10	206.3 ± 249.9	3.4 ± 2.37	220.6 ± 113.6	2	NA	1	NA	NA
		L	9	127.8 ± 153.8	4.1 ± 5.82	222.3 ± 100.1	3	NA	5	NA	NA
		O	33	936.7 ± 824.8	9.9 ± 8.11	177.0 ± 95.8	14	NA	10	NA	NA
Chen et al. [20]	2016	R	44	96.9 ± 83.2	3.5 ± 1.9	176.8 ± 54.3	0	24.2 ± 13.3	NA	44	43
		L	21	326.2 ± 368.7	5.5 ± 3.0	232.3 ± 85.4	0	21.4 ± 7.1	NA	21	20
		O	73	848.6 ± 666.9	9.7 ± 6.4	287.2 ± 144.0	2	25.9 ± 13.2	NA	70	65

*R* robot, *L* laparoscopic, *O* laparotomy, *EBL* estimated blood loss, *LHS* length of hospital stay, *OT* operating time, *PC* postoperative complication, *PN* pelvic nodes, *PR* postoperative recurrence, *OS* overall survival, *DFS* disease-free survival, *NA* data are not available

*Mean ± SD

### Quality evaluation

The methodological quality of the eight studies was assessed by MINORS, and the scores are presented in Table 3. It could be seen that the MINORS scores of the eight studies were among 16 to 18. Items such as prospective collection of data, unbiased assessment of the study endpoint, and prospective calculation of the study size were not involved in all the eight studies, and other items involving most of the eight studies were scored 2, which indicated a good quality.

**Table 3:** Results of quality evaluation using Methodological Index for Nonrandomized Studies (MINORS)

Author	Public year	A	B	C	D	E	F	G	H	I	J	K	L	Total scores
Ye et al. [26]	2017	2	2	0	2	0	2	2	0	2	2	2	2	18
Gallotta et al. [23]	2016	2	2	0	2	0	2	2	0	2	2	2	2	18
Bellia et al. [19]	2016	2	2	0	2	0	2	2	0	2	2	0	2	16
Chen et al. [21]	2015	2	2	0	2	0	1	2	0	2	2	2	2	17
Magrina et al. [24]	2011	2	2	0	2	0	2	2	0	2	2	2	2	18
Feuer et al. [22]	2013	2	2	0	2	0	2	2	0	2	2	1	2	17
Magrina et al. [25]	2013	2	2	0	2	0	1	2	0	2	2	2	2	17
Chen et al. [20]	2016	2	2	0	2	0	2	2	0	2	2	1	2	17

*A* a clearly stated aim, *B* inclusion of consecutive patients, *C* prospective collection of data, *D* endpoints appropriate to the aim of the study, *E* unbiased assessment of the study endpoint, *F* follow-up period appropriate to the aim of the study, *G* loss to follow-up less than 5%, *H* prospective calculation of the study size, *I* an adequate control group, *J* contemporary groups, *K* baseline equivalence of groups, *L* adequate statistical analyses

### Meta-analysis

#### Comparison of robotic surgery and laparoscopy

Five studies reported the variable EBL of the robotic surgery group and laparoscopy group, and there was a significant heterogeneity between studies (*P* = 0.07, *I*^2^ = 54.7%). Similarly, there was a remarkable heterogeneity between studies for the variables LHS (*P* = 0.01, *I*^2^ = 69.4%) and OT (*P* < 0.01, *I*^2^ = 70.2%). Hence, the random effects model was selected to assess the effect size of EBL, LHS, and OT. Meanwhile, there was no obvious heterogeneity between studies for the variables OS rate, DFS rate, PC, PN and PR, and the fixed effects model was selected.

**Fig. 2:**
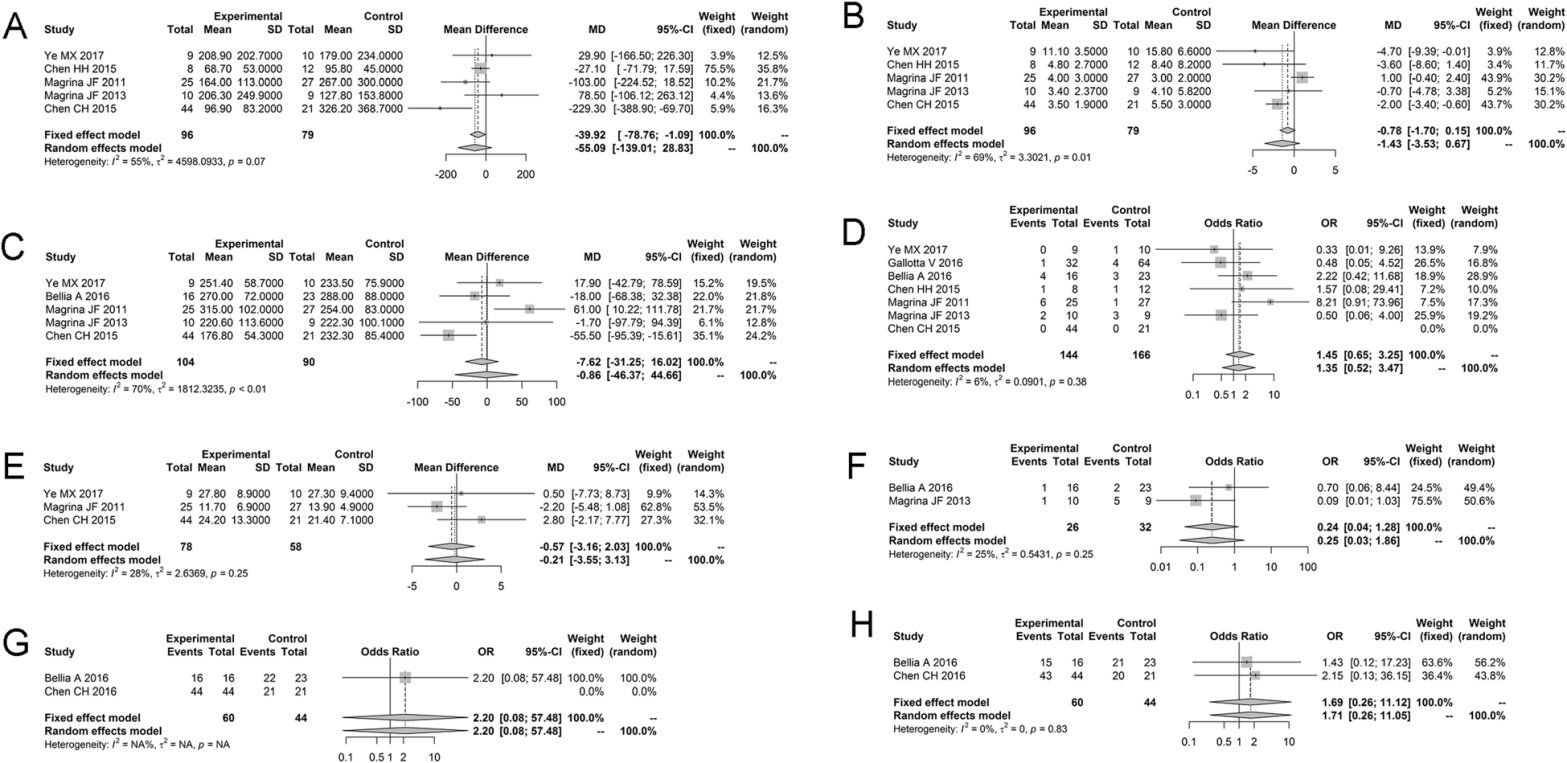
The comparisons of six variables between the robotic surgery group and laparoscopy group presented by forest plot. **a** Estimated blood loss. **b** Length of hospital stay. **c** Operating time. **d** Pelvic nodes. **e** Postoperative complication. **f** Postoperative recurrence. **g** Overall survival rate. **h** Disease-free survival rate. Each study is presented by name of the first author and year of publication; the horizontal line represents the confidence interval of each included study; the rhombus represents the pooled effect size of multiple studies; the square represents the position of the OR value

The results of the pooled estimate for each variable are shown in Table 4. It could be seen that there was no significant difference among the robotic surgery and laparoscopy groups for all variables EBL (WMD = − 55.0871, 95% CI − 139.0087; 28.8345, *P* = 0.1983), LHS (WMD = − 1.4296, 95% CI − 3.5326, 0.6734, *P* = 0.1827), OT (WMD = − 0.856, 95% CI − 46.3735; 44.6612, *P* = 0.9706), PC (OR = 1.454, 95% CI 0.6502; 3.2516, *P* = 0.3619), PN (WMD = − 0.5664, 95% CI − 3.1615; 2.0288, *P* = 0.6688), PR (OR = 0.2387, 95% CI 0.0445; 1.2792, *P* = 0.0944), OS rate (OR = 2.2000, 95% CI 0.0842; 57.4835, *P* = 0.6360), and DFS rate (OR = 1.6909, 95% CI 0.2572; 11.1175, *P* = 0.5850). The results indicated the same effect of robotic surgery and laparoscopy in the treatment of ovarian cancer (Fig. 2).

**Table 4:** Results of the meta-analysis

Variable	Sample size		Test of association			Model	Test of heterogeneity^a,b^			Egger’s test^c^	
	N1	N2	WMD/OR (95% CI)	*Z*	*P*		*Q*	*P*	*I*^*2*^ (%)	*t*	*P*
R vs. L											
EBL	96	79	− 55.0871 [− 139.0087; 28.8345]	1.29	0.1983	R	8.83	0.07	54.7	0.3887	0.7234
LHS	96	79	− 1.4296[− 3.5326; 0.6734]	1.33	0.1827	R	13.06	0.01	69.4	0.9334	0.4195
OT	104	90	− 0.8561[− 46.3735; 44.6612]	0.04	0.9706	R	13.4	< 0.01	70.2	0.7602	0.5024
PC	144	166	1.4541 [0.6502; 3.2516]	0.91	0.3619	F	5.34	0.38	6.3	1.5330	0.2001
PN	78	58	− 0.5664 [− 3.1615; 2.0288]	0.43	0.6688	F	2.78	0.25	28.2	0.7882	0.5751
PR	26	32	0.2387 [0.0445; 1.2792]	1.67	0.0944	F	1.34	0.25	25.5	NA	NA
OS rate	60	44	2.2000 [0.0842; 57.4835]	0.47	0.6360	NA	NA	NA	NA	NA	NA
DFS rate	60	44	1.6909 [0.2572; 11.1175]	0.55	0.5850	F	0.05	0.83	0.0	NA	NA
R vs. O											
EBL	125	274	− 521.7027 [− 809.7816; − 233.6238]	3.55	0.0004	R	82.54	< 0.01	93.9	1.1403	0.3178
LHS	159	274	− 5.2225 [− 6.1485; − 4.2965]	11.05	< 0.0001	F	8.36	0.14	40.2	0.1786	0.8669
OT	151	259	9.8527 [− 57.0904; 76.7958]	0.29	0.7730	R	59.75	< 0.01	93.3	0.5645	0.6119
PC	159	274	0.4710 [0.2537; 0.8747]	2.38	0.0171	F	1.77	0.88	0.0	6.8290	0.0024
PN	141	226	0.0048 [− 1.9418; 1.9514]	<0.01	0.9962	F	4.77	0.19	37.1	0.2131	0.8510
PR	73	59	0.8506 [0.3356; 2.1562]	0.34	0.7332	F	1.71	0.19	41.7	NA	NA
OS rate	107	99	6.4355 [1.6722; 24.7678]	2.71	0.0070	F	0.09	0.76	0.0	NA	NA

*WMD* weighted mean difference, *OR* odds ratio, *CI* confidence interval, *EBL* estimated blood loss, *LHS* length of hospital stay, *OT* operating time, *PC* postoperative complication, *PN* pelvic nodes, *PR* postoperative recurrence, *OS* overall survival, *DFS* disease-free survival, *NA* data are not available

^a^Random effects model was used when the *P* for heterogeneity test < 0.05; otherwise, the fixed effect model was used

^b^*P* < 0.05 is considered statistically significant for *Q* test

^c^Egger’s test to evaluate publication bias, *P* < 0.05 is considered statistically significant

#### Comparison of robotic surgery and laparotomy

Six studies reported the variable EBL of the robotic surgery and laparotomy groups, and there was a significant heterogeneity between studies (*P* < 0.01, *I*^2^ = 93.9%). Similarly, there was a remarkable heterogeneity between studies for the variable OT (*P* < 0.01, *I*^2^ = 93.3%). Therefore, the random effects model was selected to assess the effect of EBL and OT. Meanwhile, no obvious heterogeneity was identified between studies for the variables OS rate, LHS, PC, PN, and PR, and the fixed effects model was selected.

**Fig. 3:**
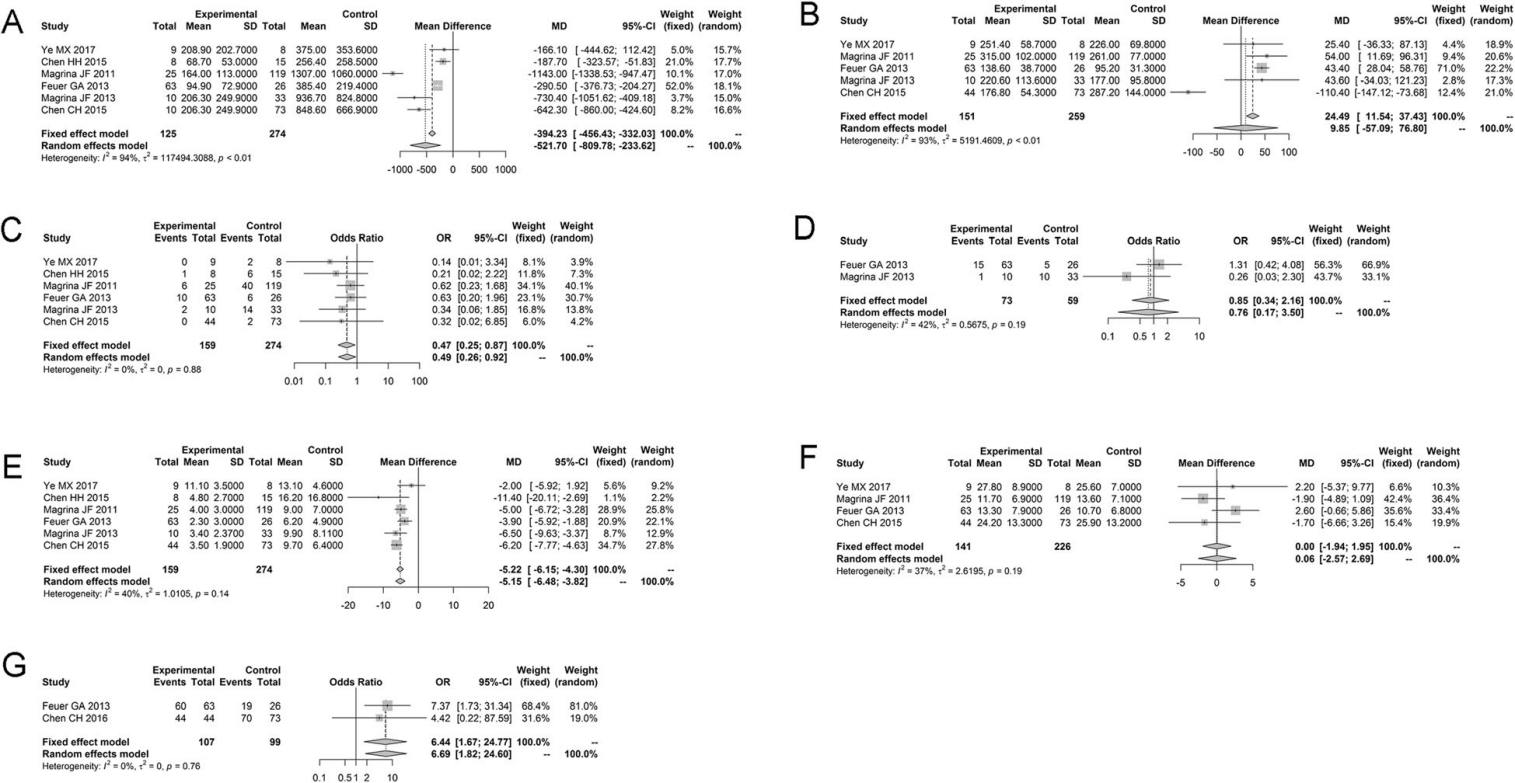
The comparisons of six variables between the robotic surgery group and laparotomy group presented by forest plot. **a** Estimated blood loss. **b** Operating time. **c** Pelvic nodes. **d** Postoperative recurrence. **e** Length of hospital stay. **f** Postoperative complication. **g** Overall survival rate. Each study is presented by name of the first author and year of publication; the horizontal line represents the confidence interval of each included study; the rhombus represents the pooled effect size of multiple studies; the square represents the position of the OR value

The results of the pooled estimate for each variable are shown in Table 4, and it revealed that there was no statistical difference among the robotic surgery and laparotomy groups for the variables OT (WMD = 9.8527, 95% CI − 57.0904; 76.7958, *P* = 0.7730), PN (WMD = 0.0048, 95% CI − 1.9418; 1.9514, *P* = 0.9962), and PR (OR = 0.8506, 95% CI 0.3356; 2.1562, *P* = 0.7332), indicating that the robotic surgery and laparotomy in the treatment of ovarian cancer had similar effect on OT, PN, and PR. Only one study reported the variable DFS rate of the robotic surgery and laparotomy groups, and no statistical difference was found (data not shown). Meanwhile, there was a significant difference among the robotic surgery and laparotomy groups for the variables EBL (WMD = − 521.7027, 95% CI − 809.7816; − 233.6238, *P* = 0.0004), LHS (WMD = − 5.2225, 95% CI − 6.1485; − 4.2965, *P* < 0.0001), PC (OR = 0.4710, 95% CI 0.2537; 0.8747, *P* = 0.0171), and OS rate (OR = 6.4355, 95% CI 1.6722; 24.7678, *P* = 0.0070), presenting lesser EBL, shorter LHS, lower PC, and higher OS rate with robotic surgery treatment (Fig. 3).

### Publication bias and sensitivity analysis

A significant publication bias (*t* = 6.8290, *P* = 0.002405) was identified for the variable PC in the robotic surgery and laparotomy groups, but no publication bias was identified for the other variables not only in the robotic surgery and laparotomy groups, but also in the robotic surgery and laparoscopy groups, suggesting the results of these indicators were reliable (Table 4).

Sensitivity analysis revealed that there was a difference between random effects model (*P* > 0.05) and fixed effects model (*P* < 0.05) for the pooled effect values of variables EBL and OT in the robotic surgery and laparoscopy groups. The pooled effect values of other variables based on random effects model and fixed effects model were consistent with each other, indicating that the results of those variables were stable (Table 5).

**Table 5:** Results of sensitivity analysis

Variable	Group	Effect model	*K*	WMD/OR [95% CI]	*Z*	*P*
Estimated blood loss	R vs. L	Fixed	5	− 39.9247 [− 78.7622; − 1.0871]	2.01	0.0439
	R vs. L	Random	5	− 55.0871 [− 139.0087; 28.8345]	1.29	0.1983
	R vs. O	Fixed	6	− 394.2279 [− 456.4271; − 332.0286]	12.42	< 0.0001
	R vs. O	Random	6	− 521.7027 [− 809.7816; − 233.6238]	3.55	0.0004
Length of hospital stay	R vs. L	Fixed	5	− 0.7770 [− 1.7023; 0.1484]	1.65	0.0998
	R vs. L	Random	5	− 1.4296 [− 3.5326; 0.6734]	1.33	0.1827
	R vs. O	Fixed	6	− 5.2225 [− 6.1485; − 4.2965]	11.05	< 0.0001
	R vs. O	Random	6	− 5.1505 [− 6.4793; − 3.8216]	7.60	< 0.0001
Operating time	R vs. L	Fixed	5	− 7.6177 [− 31.2545; 16.0192]	0.63	0.5276
	R vs. L	Random	5	− 0.8561 [− 46.3735; 44.6612]	0.04	0.9706
	R vs. O	Fixed	5	24.4862 [11.5380; 37.4344]	3.71	0.0002
	R vs. O	Random	5	9.8527 [− 57.0904; 76.7958]	0.29	0.7730
Postoperative complication	R vs. L	Fixed	6	1.4541 [0.6502; 3.2516]	0.91	0.3619
	R vs. L	Random	6	1.3484 [0.5235; 3.4731]	0.62	0.5357
	R vs. O	Fixed	6	0.4710 [0.2537; 0.8747]	2.38	0.0171
	R vs. O	Random	6	0.4878 [0.2600; 0.9154]	2.24	0.0254
Pelvic nodes	R vs. L	Fixed	3	− 0.5664 [− 3.1615; 2.0288]	0.43	0.6688
	R vs. L	Random	3	− 0.2074 [− 3.5497; 3.1349]	0.12	0.9032
	R vs. O	Fixed	4	0.0048 [−1.9418; 1.9514]	<0.01	0.9962
	R vs. O	Random	4	0.0640 [− 2.5658; 2.6937]	0.05	0.9620
Postoperative recurrence	R vs. L	Fixed	2	0.2387 [0.0445; 1.2792]	1.67	0.0944
	R vs. L	Random	2	0.2463 [0.0326; 1.8617]	1.36	0.1745
	R vs. O	Fixed	2	0.8506 [0.3356; 2.1562]	0.34	0.7332
	R vs. O	Randomed	2	0.7633 [0.1665; 3.4995]	0.35	0.7281
Overall survival rate	R vs. L	Fixed	2	2.2000 [0.0842;5 7.4835]	0.47	0.6360
	R vs. L	Random	2	2.2000 [0.0842; 57.4835]	0.47	0.6360
	R vs. O	Fixed	2	6.4355 [1.6722; 24.7678]	2.71	0.0070
	R vs. O	Randomed	2	6.6854 [1.8172; 24.5959]	2.86	0.0040
Disease-free survival rate	R vs. L	Fixed	2	1.6909 [0.2572; 11.1175]	0.55	0.5850
	R vs. L	Randomed	2	1.7085 [0.2641; 11.0547]	0.56	0.5740

*K* the number of articles included, *R* robot, *L* laparoscopic, *O* laparotomy, *WMD* weighted mean difference, *OR* odds ratio

## Discussion

In the current study, the effect of robotic surgery in ovarian cancer therapy was compared with laparoscopy and laparotomy based on 647 patients recorded in eight studies. The results of the meta-analysis revealed that the robotic surgery and laparoscopy had the same effect in ovarian cancer therapy, while the robotic surgery had lesser EBL, shorter LHS, lower PC, and higher OR rate compared with laparotomy in ovarian cancer therapy.

Robotic system provided some merits, including 3-D visualization, ergonomic position, and eliminating hand tremor, promising a more accurate operation in various complex tumor surgery [9]. Reportedly, robotic surgery was viable in the staging for early ovarian cancer and had no obvious difference with laparoscopy in clinical outcomes including EBL, PC, and PN [23, 27]. Zapardiel et al. demonstrated that the robotic surgery presented similar outcomes of blood loss, PC, and LHS with laparoscopy in ovarian remnant syndrome therapy [28]. Moreover, a previous study had showed that the robotic surgery and laparoscopy were all safe and feasible for hepatectomies with no significant difference in surgical outcomes, except for the minimal incision of robotic surgery [29]. In accord with those views, our study revealed that robotic surgery provided a similar effect with laparoscopy in ovarian cancer therapy.

Compared with laparotomy, robotic surgery provided some advantages in the treatment of primary or recurrent ovarian cancer, including lesser EBL and shorter length of stay [30]. A previous study had reported it was advisable to select robotic surgery for the endometrial cancer staging than laparotomy surgical, with a decrease of hospital stay and EBL as well as PC rates and an increase of BMI [31]. Previous study had revealed that robotic surgery and laparoscopy presented a similar effect in radical hysterectomy and ovarian remnant syndrome and were more advisable versus laparotomy with a decrease of blood loss and hospital stay and PC [28, 32]. In addition, Subramaniam et al. proved that robotic surgery provided an improvement of surgical outcomes and might be a more advisable approach for obese women with endometrial cancer [33]. Those findings were all consistent with the results of our meta-analysis; hence, we indicated that robotic surgery was secure and feasible in ovarian cancer treatment and provided several merits than laparotomy.

Robotic surgery was considered as a promising new technique which could be used to optimize clinical management of patients with gynecological carcinoma. Nevertheless, concerns had been expressed focusing on the cost of robotic surgery compared to laparoscopy or laparotomy [34], and different opinions were proposed. Lindfors et al. suggested that there was no statistically significant difference in costs between robotic surgery and laparotomy in the treatment of elderly patients with endometrial cancer [35]. Novellis et al. indicated that the hospital could make a profit in spite of the high cost of robotic surgery [36]. A study reported that comparable outcomes were observed between robotic surgery and laparoscopy in the treatment for colon cancer, but robotic surgery showed higher treatment cost [37]. Besides, it was reported in two reviews that the high cost was mainly reflected in the acquisition, maintenance of instruments, and training of medical personnel using the robotic system [34, 38]. Further studies were recommended in the future to reduce the cost of robotic operations.

The heterogeneity test showed an obvious heterogeneity among studies for few variables, and we selected the random effect model to assess the pooled effect. Several reasons were considered as follows: (1) differences between different countries, race, and regions, such as China, Italy, and the USA; (2) differences among ovarian cancer with different types and stages, such as early stage of ovarian cancer, stage IA–IIIC epithelial ovarian cancer, recurrent ovarian cancer, and epithelial ovarian cancer; (3) influences from confounding factors such as gender, age, and other demographic data; (4) the same result might be interpreted differently by different authors, which might lead to a misleading result.

In this study, the meta-analysis was used to comprehensively compare the effect of robotic surgery in the treatment of ovarian cancer with laparotomy and laparoscopy, and we found that robotic surgery presented several merits in ovarian cancer therapy compared with laparotomy. But there still retained some limitations: (1) Subgroup analysis and meta-regression analysis for tumor stages, time of follow-up, age, and race were not performed due to the incomplete demographic data and less included studies. (2) There was a significant publication bias for the variable PC in the robotic surgery and laparotomy groups which might affect the result. In addition, the publication bias for the variable PR was not detected due to less included studies. (3) The result of sensitivity analysis showed that the pooled effect of the variables EBL (robotic surgery and laparoscopy groups) and OT (robotic surgery and laparotomy groups) was inconsistent under random or fixed effects models, which indicated the comparison results of the two variables were unstable. (4) Last but not the least, the related data were incomplete in the included studies, and only one study contained the related data of all the six variables, such as EBL, LHS, and OT were not mentioned in two studies; PN was not mentioned in four studies; PR and OS rate were not mentioned in five studies; and DFS rate was not mentioned in six studies. Besides, some of the data in several literatures were skewed, but these literatures also provide mean ± SD data which met the requirements of a meta-analysis, and they were included in this meta-analysis. Hence, an analysis based on more studies with high quality was needed to confirm the clinical effect of robotic surgery in ovarian cancer treatment.

## Conclusions

In the current meta-analysis, the robotic surgery and laparoscopy presented the same effect in the treatment of ovarian cancer, while the robotic surgery provided lesser EBL, LHS, and PC and higher OS rate versus laparotomy. However, it failed to show oncological safety and recurrence by pathological stages or histologic types in this meta-analysis, and those confounding factors might affect the clinical outcome. Future meta-analyses with a larger number of eligible randomized controlled trial studies were needed to determine the most suitable treatment method for patients with different types and stages of ovarian cancer.

## Additional file

Additional file 1: **Table S1.** The detailed retrieval strategy in PubMed database. (DOCX 12 kb)

## Data Availability

All data generated or analyzed during this study are included in this published article.

## Abbreviations

BMI: Body mass index
CI: Confidence interval
DFS: Disease-free survival
EBL: Estimated blood loss
LHS: Length of hospital stay
MINORS: Methodological Index for Nonrandomized Studies
OR: Odds ratio
OS: Overall survival
OT: Operating time
PC: Postoperative complication
PN: Pelvic nodes
PR: Postoperative recurrence
WMD: Weighted mean difference
