# Supplementary material for: Comparison of efficacy of robotic surgery, laparoscopy, and laparotomy in the treatment of ovarian cancer: a meta-analysis
Source: World J Surg Oncol. 2019 Sep 13;17:162. doi: 10.1186/s12957-019-1702-9 (PMC6743103; doi:10.1186/s12957-019-1702-9)
Supplement: Supplementary file 1 — Table S1. The detailed retrieval strategy in PubMed database. (DOCX 12 kb) [file 12957_2019_1702_MOESM1_ESM.docx]

**The detailed retrieval strategy in PubMed database.**

Search details: ((robot[All Fields] OR robot'[All Fields] OR robot'consists[All Fields] OR robot'electronically[All Fields] OR robot's[All Fields] OR robota[All Fields] OR robotac[All Fields] OR robotach[All Fields] OR robotakh[All Fields] OR robotanalyst[All Fields] OR robotanalyst's[All Fields] OR robotarium[All Fields] OR robotassisted[All Fields] OR robotassistedlaparoscopic[All Fields] OR robotassisteret[All Fields] OR robotassistirovannaia[All Fields] OR robotchirurgie[All Fields] OR robotdalen[All Fields] OR robotdoc2k[All Fields] OR robote[All Fields] OR robotechnologist[All Fields] OR roboteeg[All Fields] OR robotek[All Fields] OR robotem[All Fields] OR robotene[All Fields] OR roboter[All Fields] OR roboterarm[All Fields] OR roboterarmes[All Fields] OR roboterassistenz[All Fields] OR roboterassistiert[All Fields] OR roboterassistierte[All Fields] OR roboterassistierten[All Fields] OR roboterassistierter[All Fields] OR roboterassistiertes[All Fields] OR roboterchirurgie[All Fields] OR robotereinsatz[All Fields] OR robotergefuhrte[All Fields] OR robotergefuhrten[All Fields] OR robotergesteuerte[All Fields] OR robotergesteuerten[All Fields] OR robotergestutzte[All Fields] OR robotergestutztem[All Fields] OR robotergestutzten[All Fields] OR robotergestutzter[All Fields] OR robotergestutztes[All Fields] OR roboterpersonlichkeiten[All Fields] OR roboterpraparation[All Fields] OR roboterprogramms[All Fields] OR roboters[All Fields] OR robotersystem[All Fields] OR robotersysteme[All Fields] OR robotersystems[All Fields] OR robotertechniken[All Fields] OR robotertechnologie[All Fields] OR roboterunterstutzt[All Fields] OR roboterunterstutzte[All Fields] OR roboterunterstutzten[All Fields] OR roboterunterstutzter[All Fields] OR roboterunterstutzung[All Fields] OR robotewskyj[All Fields] OR robotex[All Fields] OR robotfoto[All Fields] OR robotgeassisteerde[All Fields] OR robotham[All Fields] OR robotherapist[All Fields] OR robotherapist'[All Fields] OR robotherapy[All Fields] OR roboti[All Fields] OR robotic[All Fields] OR robotic'[All Fields] OR robotica[All Fields] OR robotical[All Fields] OR robotically[All Fields] OR roboticallyassisted[All Fields] OR roboticapproach[All Fields] OR roboticas[All Fields] OR roboticassisted[All Fields] OR roboticbed[All Fields] OR roboticheskoi[All Fields] OR roboticians[All Fields] OR roboticist[All Fields] OR roboticists[All Fields] OR roboticists'[All Fields] OR roboticization[All Fields] OR roboticized[All Fields] OR roboticizes[All Fields] OR roboticka[All Fields] OR robotickai[All Fields] OR roboticke[All Fields] OR robotickeho[All Fields] OR robotickou[All Fields] OR roboticky[All Fields] OR robotickych[All Fields] OR robotickym[All Fields] OR robotico[All Fields] OR roboticos[All Fields] OR roboticpancreatoduodenectomy[All Fields] OR roboticprogramme[All Fields] OR robotics[All Fields] OR robotics'[All Fields] OR robotics,[All Fields] OR roboticslab[All Fields] OR roboticsuniversity[All Fields] OR roboticsurgery[All Fields] OR roboticsystem[All Fields] OR roboties[All Fields] OR robotik[All Fields] OR robotika[All Fields] OR robotikai[All Fields] OR robotiker[All Fields] OR robotikzentren[All Fields] OR robotin[All Fields] OR robotiq[All Fields] OR robotique[All Fields] OR robotiques[All Fields] OR robotiquette[All Fields] OR robotiquette'[All Fields] OR robotis[All Fields] OR robotisation[All Fields] OR robotisch[All Fields] OR robotische[All Fields] OR robotisches[All Fields] OR robotise[All Fields] OR robotised[All Fields] OR robotisee[All Fields] OR robotisees[All Fields] OR robotisert[All Fields] OR robotises[All Fields] OR robotitian[All Fields] OR robotix[All Fields] OR robotizacion[All Fields] OR robotizada[All Fields] OR robotizado[All Fields] OR robotizados[All Fields] OR robotization[All Fields] OR robotizatsii[All Fields] OR robotize[All Fields] OR robotized[All Fields] OR robotizing[All Fields] OR robotizirovannoi[All Fields] OR robotizirovannom[All Fields] OR robotizirovannykh[All Fields] OR robotizzata[All Fields] OR robotizzato[All Fields] OR robotka[All Fields] OR robotkeztechnika[All Fields] OR robotkirurgi[All Fields] OR robotkirurgisk[All Fields] OR robotkova[All Fields] OR robotless[All Fields] OR robotlike[All Fields] OR robotmajsebeszet[All Fields] OR robotmanipulators[All Fields] OR robotmcu[All Fields] OR robotmd[All Fields] OR robotmediated[All Fields] OR robotn'ikov[All Fields] OR robotnakov[All Fields] OR robotnam[All Fields] OR robotni[All Fields] OR robotnic[All Fields] OR robotnicza[All Fields] OR robotnicze[All Fields] OR robotniczej[All Fields] OR robotniczo[All Fields] OR robotniczych[All Fields] OR robotniczym[All Fields] OR robotnik'ow[All Fields] OR robotnika[All Fields] OR robotnikami[All Fields] OR robotnikinin[All Fields] OR robotnikov[All Fields] OR robotnikow[All Fields] OR robotnitsi[All Fields] OR robotnon[All Fields] OR robotnykh[All Fields] OR robotnykiv[All Fields] OR roboto[All Fields] OR robotocs[All Fields] OR robotoi[All Fields] OR robotoiu[All Fields] OR robotok[All Fields] OR robotol[All Fields] OR robotosposobnost[All Fields] OR robotosposobnost'[All Fields] OR robototekhnika[All Fields] OR robototekhnika'[All Fields] OR robototekhniki[All Fields] OR robototherapy[All Fields] OR robotow[All Fields] OR robotrac[All Fields] OR robotrat[All Fields] OR robotreviewer[All Fields] OR robotreviewer's[All Fields] OR robotripping[All Fields] OR robotrobot[All Fields] OR robotron[All Fields] OR robots[All Fields] OR robots'[All Fields] OR robots'designs[All Fields] OR robotsci[All Fields] OR robotscientist[All Fields] OR robotsebeszet[All Fields] OR robotsebeszeti[All Fields] OR robotsensor[All Fields] OR robotsfor[All Fields] OR robotta[All Fields] OR robotter[All Fields] OR robotti[All Fields] OR robottiavusteinen[All Fields] OR robotto[All Fields] OR robottom[All Fields] OR robottrade[All Fields] OR robotu[All Fields] OR robotuna[All Fields] OR robotutor[All Fields] OR robotworld[All Fields] OR roboty[All Fields] OR robotyka[All Fields] OR robotyki[All Fields]) OR "Da Vinci"[All Fields] OR Leonardo's[All Fields]) AND ("ovarian cancer"[All Fields] OR oophoroma[All Fields] OR "carcinoma of ovary"[All Fields] OR "ovarian carcinoma"[All Fields] OR "Ovary cancer"[All Fields]) AND (("laparoscopy"[MeSH Terms] OR "laparoscopy"[All Fields]) OR ("laparoscopy"[MeSH Terms] OR "laparoscopy"[All Fields] OR "laparoscopic"[All Fields]) OR ("laparotomy"[MeSH Terms] OR "laparotomy"[All Fields]) OR "Open surgery"[All Fields])
